# A Report of a Case of Segmental Zoster Paresis Demonstrated by Limb Paralysis and a Review of the Literature

**DOI:** 10.7759/cureus.45691

**Published:** 2023-09-21

**Authors:** Luis F Castro, Siddharth A Atwal, Jessica M Ramirez, Jali Garza, Judy Lalmuanpuii

**Affiliations:** 1 Department of Internal Medicine, Texas Tech University Health Sciences Center, Lubbock, USA; 2 Department of Internal Medicine, University of Arkansas for Medical Sciences, Little Rock, USA

**Keywords:** monoparesis, segmental zoster paresis, varicella-zoster virus, limb paralysis, shingles

## Abstract

Varicella zoster virus (VZV) lies dormant in our spinal dorsal root ganglia until reactivation occurs and causes herpes zoster. VZV can spread from the dorsal root to the neighboring ventral root and cause subsequent segmental paresis. In this case report, we present the case of a 78-year-old female who was hospitalized after she developed right upper extremity paresis and altered mental status four days after the eruption of a vesicular rash involving the same dermatome. The patient received intravenous acyclovir, gabapentin, and inpatient rehabilitation. She was found to have made a full recovery one year later. Pain and a vesicular rash is the most common presentation of VZV infection in the elderly. However, segmental zoster paresis should be suspected in any patient with paralysis and a recent diagnosis of herpes zoster.

## Introduction

Primary infection with varicella zoster virus (VZV) in non-immune infants causes chickenpox, also known as varicella [1]. After primary infection, VZV can lay dormant in the dorsal root ganglia of the spinal cord and cranial nerve ganglia [2]. VZV found along sensory fibers can reactivate later in life and cause herpes zoster (HZ) or shingles infection, resulting in a painful vesicular eruption along a dermatomal segment [1]. The estimated incidence is about 3 to 4 per 1,000 person-years, and this estimate increases dramatically for people over 80 years of age [2]. HZ infection can have a prodromal phase of pain and itchiness before the classic vesicular rash develops. The rash can last two to four weeks before it scabs and heals. Postherpetic neuralgia is the most common complication of HZ and develops in approximately 5% to 20% of patients [2]. Reactivation of dormant VZV and inflammatory response can damage sensory and central neurons, leading to allodynia and postherpetic neuralgia. Other complications from HZ include encephalitis, segmental zoster paresis (SZP), pneumonia, and death [3].

SZP is another form of neuropathy resulting from VZV reactivation, and it has been found to occur in around 0.5% of HZ patients [4]. VZV can spread from the dorsal root to the neighboring ventral root and cause subsequent motor weakness. This usually occurs in the same anatomical region as the presenting rash, but the two can rarely be dissociated. Treatment usually includes antivirals, steroids, and physical therapy [5]. Early antiviral treatment improves the chances of achieving full motor recovery. Most patients who develop SZP have an excellent prognosis and will recover motor function after six months to a year [6]. In this article, we will present the case of a 78-year-old female diagnosed with SZP after she presented with altered mental status (AMS) and single-limb monoparesis.

## Case presentation

A 78-year-old Caucasian female with a medical history of rheumatoid arthritis on immunosuppressive therapy, metabolic syndrome, and chronic kidney disease was admitted to the hospital for AMS and right arm weakness. Three days before admission, she was seen in the outpatient setting for a dermatomal vesicular eruption of the right arm with onset four days prior, which was diagnosed as HZ. She was prescribed valacyclovir and gabapentin. Physical examination in the emergency department revealed a confused and lethargic patient, without cranial nerve deficits, diminished strength on her right upper extremity (RUE), but intact deep tendon reflexes. On intake labs, she was found to have hypotonic hyponatremia (119 mmol/L). Initial radiologic studies showed no acute intracranial hemorrhage, masses, or ventriculomegaly. She was admitted to the medical intensive care unit (ICU) for correction of hyponatremia and concerns for viral meningitis and metabolic encephalopathy.

**Figure 1:**
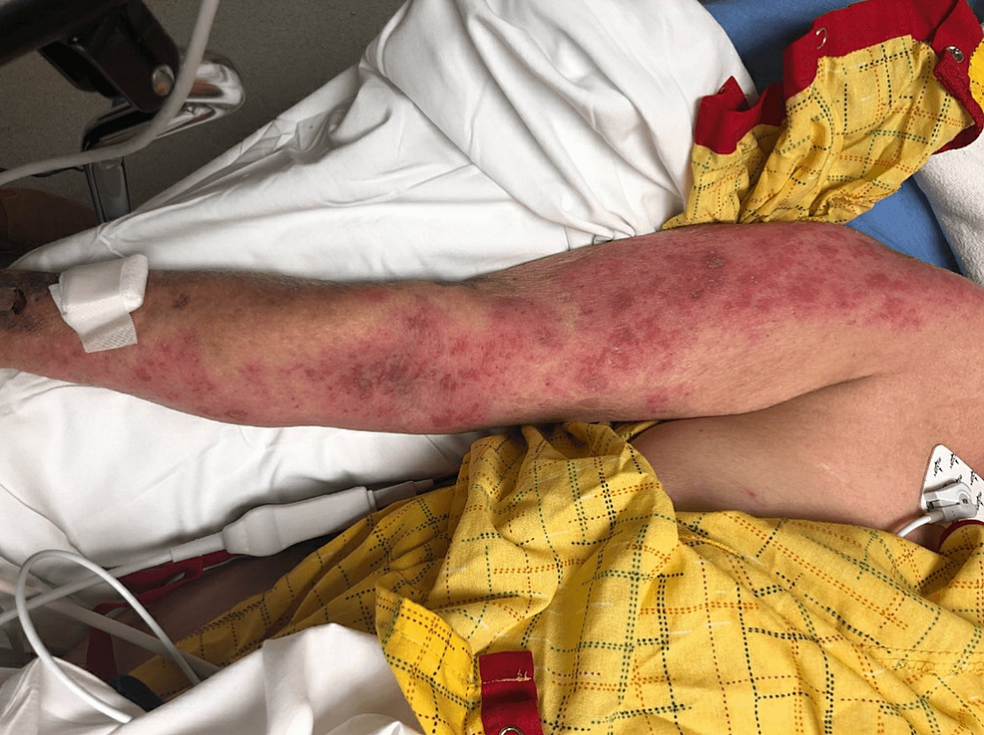
Herpes zoster vesicular rash of the right upper extremity at admission.

After transferring to the ICU, magnetic resonance imaging (MRI) of the brain showed no acute infarction or intracranial masses. Lumbar puncture showed normal opening pressure (10 mm H_2_O), and cerebrospinal fluid analysis studies were remarkable for elevated protein (138 mg/dL) and white blood count (35 per mm^3^) with lymphocytic predominance but normal glucose level. These raised suspicions of viral meningitis, and the patient was started on intravenous (IV) acyclovir. Shoulder, arm, and forearm radiographs were negative for any fractures. During the ICU course, physical examination was notable for somnolence, RUE weakness (3/5), and a painful vesicular rash (Figure 1). After correcting sodium levels with hypertonic saline and fluid restriction, the patient was transferred to the intermediate unit for further care.

Despite correcting sodium levels, the patient continued to be somnolent on examination. A neurological physical exam was notable for isolated RUE weakness (3/5) and diminished sensation to temperature, pain, and vibration. Deep tendon reflexes were also diminished (1+) on the RUE. Neurology was subsequently consulted for RUE weakness. Cervical spine MRI was positive for degenerative disc changes but negative for evidence of compressive myelopathy. Thus, a diagnosis of SZP was given, and a continued course of IV acyclovir for 21 days was recommended. After antiviral treatment, the rash subsided, but the RUE weakness persisted. Physical therapy recommended discharging the patient to an inpatient rehabilitation facility where she could continue with physical therapy exercises for about two weeks. The patient was seen again one year later and was noted to have completely recovered from RUE weakness.

## Discussion

In this article, we have presented a rare case of HZ complicated by SZP in an immunosuppressed elderly woman with a long-standing history of rheumatoid arthritis. HZ is a common issue in the geriatric population. Some studies estimate that one in three adults will develop HZ during their lifetime [7]. Most cases of HZ present with a painful vesicular rash along a dermatomal segment. However, a few may experience a disease course complicated by cranial nerve involvement, such as in herpes zoster ophthalmicus and oticus. Postherpetic neuralgia, a form of mononeuropathy, may result from damage to peripheral and central neurons due to HZ infection and the body’s inflammatory response to infection. Damage to ventral horn neurons in the spinal cord or motor efferent fibers can lead to SZP, another form of residual mononeuropathy. Other rare complications of HZ infection include encephalitis, pneumonia, and death [3].

Our patient presented with AMS, hyponatremia, and RUE paresis. Computed tomography and MRI imaging ruled out intracranial hemorrhage and brain infarction that could explain the AMS and RUE paresis. At first, the cause of AMS was attributed to metabolic encephalopathy. Still, the recent diagnosis of HZ obscured the exact etiology of the confusion. Although uncommon, HZ could have been the cause of AMS in a patient who already suffered from SZP, an unusual complication of HZ. The lack of response to the correction of underlying hyponatremia yields credibility to the hypothesis that HZ encephalitis was the cause of her altered mentation. Instead, improvement in mentation was observed after a couple of days on IV acyclovir.

Regarding the RUE weakness, radiographs were done to rule out skeletal fractures that could explain her symptoms, and an MRI of the cervical spine was also non-contributory. No guidelines have been established to aid the clinician in diagnosing SZP, given the rarity of this condition. Electromyography (EMG) and nerve conduction studies have been used by a few published reports to aid in diagnosing SZP when the etiology of monoparesis is unclear. EMG findings may be similar to those of other axonopathies with reduced amplitude of motor or sensory action potentials [4]. However, the authors of this article opted not to perform nerve conduction studies because of the timeline of symptoms, treatment response, and the lack of evidence for alternative diagnoses. Fortunately, our patient experienced complete motor recovery one year later after a 21-day course of IV acyclovir, gabapentin, and inpatient rehabilitation. This finding is supported by our literature review, which suggests that most patients fully recover one to two years after the onset of symptoms [6,8].

Segmental zoster neuropathy

SZP is a rare complication of HZ, and it has been documented to occur in approximately 0.5% of HZ cases [3]. A summary and comparisons of literature on SZP, location affected, treatment, and outcome are summarized in Table 1. Out of the available reports, most cases of SZP occur in the immunosuppressed and the elderly, with the average age of onset at 70 years [6]. Paralysis of the affected nerve is more common in head and neck presentations, whereas weakness is most commonly seen in patients with upper extremity involvement [5,6]. A recent article by Liu and colleagues [4] found that SZP occurs more commonly in the upper extremities than lower extremities. Diagnosis of SZP is clinical, but EMG and nerve conduction studies can also be used as auxiliary tests [6]. Exclusion of alternative diagnoses such as stroke, compressive myelopathy, and meningitis or encephalitis is also important. Treatment typically consists of a 21-day course of antiviral therapy, steroids, gabapentin, and physical therapy. Most published records report full or near-complete recovery by one year after the onset of symptoms.

**Table 1:** Summary of reported cases of segmental zoster paresis F: female; M: male; LUE: left upper extremity; RLE: right lower extremity; RUE: right upper extremity; NCS/EMG: nerve conduction studies or electromyography; CXR: chest x-ray; ACV: acyclovir; VCV: valacyclovir; GCV: ganciclovir; GBP: gabapentin; PT: physical therapy.

Author	Year	Sample size	Age	Sex	Location	Diagnosis	Treatment	Outcome
Park et al. [5]	2022	1	79	F	LUE	Clinical	Steroids + nerve block	Full recovery
Chen et al. [8]	2020	1	72	M	LUE	NCS/EMG	ACV + steroids + GBP	Partial recovery
Anaya-Prado et al. [9]	2018	1	62	M	Abdomen	Clinical	ACV + GBP	Full recovery
Araz et al. [10]	2017	1	69	M	LUE	NCS/EMG	ACV + steroids	Full recovery
He et al. [11]	2018	2	67	F	Bladder	Clinical	GCV	Full recovery
			42	M	Bladder	Clinical	GCV	Full recovery
Kawajiri et al. [12]	2007	3	77	M	LUE	NCS/EMG	ACV + steroids + nerve block	Full recovery
			57	M	RLE	NCS/EMG	ACV + steroids + nerve block	Full recovery
			65	F	LUE	NCS/EMG	ACV + nerve block	Full recovery
Kim and Chung [13]	2016	1	84	M	RUE	NCS/EMG	VCV + steroids + GBP	Partial recovery
Paudyal et al. [14]	2006	1	60	M	Diaphragm	Clinical + CXR	Amitriptyline	Full recovery
Rastegar et al. [15]	2015	1	59	F	LUE	NCS/EMG	ACV + PT	Partial recovery
Ruppert et al. [16]	2010	3	10	M	LUE	NCS/EMG	ACV + PT + morphine	Full recovery
Saleem et al. [17]	2018	1	84	F	Diaphragm	NCS/EMG	ACV	Full recovery
Teo et al. [18]	2016	1	74	M	RLE	Clinical	ACV	Full recovery
Whitby and Bateman [19]	2018	1	52	M	Left foot	NCS/EMG	Conservative	Partial recovery
Yoo et al. [20]	2019	3	67	M	Abdomen	NCS/EMG	VCV + GBP	Full recovery
			68	M	Abdomen	Clinical	VCV + GBP	Partial recovery
			74	F	Abdomen	Clinical	VCV + GBP	Persistent deficit

## Conclusions

SZP is a rare complication of HZ and typically presents in elderly patients or immunosuppressed. The diagnosis of SZP should be suspected in any patient presenting with symptoms of monoparesis, particularly in the setting of a vesicular rash involving the same dermatome. However, special attention should be given to ruling out other potential causes of weakness, such as stroke. SZP is a clinical diagnosis in a patient with HZ rash and monoparesis, but EMG and nerve conduction studies can be used to aid the clinician in diagnosis. Prompt treatment of patients with SZP may reduce the duration of symptoms and the development of other complications. Like general HZ infection, treatment of SZP consists of antivirals, gabapentin, steroids, and physical therapy. The prognosis is excellent, and full recovery of motor function occurs in almost one year after the onset of symptoms.
